# Information Bottleneck for Communication-Efficient Multi-Agent Reinforcement Learning in UAV Swarms

**DOI:** 10.3390/e28080919

**Published:** 2026-08-17

**Authors:** Zheng Yang, Guohao Li, Yali Xue

**Affiliations:** College of Automation Engineering, Nanjing University of Aeronautics and Astronautics, Nanjing 211106, China; yangzz@nuaa.edu.cn (Z.Y.); 032350224@nuaa.edu.cn (G.L.)

**Keywords:** information bottleneck, multi-agent reinforcement learning, UAV swarms, Cauchy–Schwarz divergence, communication-efficient learning, cooperative control

## Abstract

Multi-agent reinforcement learning has emerged as a promising paradigm for cooperative unmanned aerial vehicle (UAV) swarm coordination. However, existing communication-aware MARL methods primarily focus on communication topology, message routing, and message aggregation, while the information content of the exchanged messages is often only implicitly controlled. In realistic UAV networks, inter-agent communication is constrained by limited bandwidth, communication range, energy consumption, and packet loss. It is therefore desirable for each UAV to transmit compact and task-relevant information rather than dense and redundant latent features. In this paper, we propose IB-CEMARL, an information-bottleneck-guided, communication-efficient multi-agent reinforcement learning framework for UAV swarms. We formulate inter-UAV communication as a minimal sufficient message-learning problem in which each UAV encodes its local observation into a stochastic bottleneck message before exchanging information with its neighbors. Cauchy–Schwarz divergence-based quadratic mutual information is adopted as a unified dependence measure to jointly regularize message compression, preserve decision-relevant information, and reduce statistical redundancy among neighboring UAV messages. Extensive experiments demonstrate that IB-CEMARL achieves superior cooperative performance, reduced message redundancy, and stronger robustness compared with representative communication-aware MARL baselines. In particular, IB-CEMARL improves the average return by 4.9% and reduces inter-message dependence by 29.0% compared with the KL-IB-MARL baseline while maintaining efficient communication under constrained bandwidth settings.

## 1. Introduction

Unmanned aerial vehicle (UAV) swarms have emerged as a promising platform for time-critical and large-scale missions, such as disaster response, wildfire monitoring, environmental sensing, communication relay, and infrastructure inspection. A prominent application is to use autonomous UAVs as rapidly deployable aerial relays when terrestrial communication infrastructure is damaged by natural disasters or overloaded during crowded events [1,2]. In scenarios such as wildfire monitoring, post-disaster search and rescue, and remote-area surveillance, UAVs may also operate beyond the direct control radius of human operators, requiring on-board autonomy and peer-to-peer coordination. Compared with a single UAV, a cooperative swarm can cover larger areas, provide more robust sensing, and adapt more flexibly to dynamic environments. However, these advantages rely critically on effective coordination among multiple UAVs. Since each UAV usually has only partial observations of the environment and limited knowledge of other UAVs’ states, inter-agent communication becomes essential for cooperative decision-making in UAV swarms [3,4,5].

Multi-agent reinforcement learning (MARL) provides a natural framework for the learning of cooperative policies under partial observability and dynamic interactions [6,7,8]. Recent UAV-oriented MARL studies further demonstrate its effectiveness in multi-UAV collaboration networks, ground surveillance, UAV-aided edge computing, and cooperative collision avoidance [9,10,11,12]. In particular, centralized training with decentralized execution (CTDE) has become a widely adopted paradigm, where agents can exploit global information during training while executing decentralized policies based on local observations at test time [6,7,8,13]. For UAV swarms, this paradigm is especially attractive because decentralized execution avoids a single point of failure and better matches the operational constraints of real-world aerial networks. Recent communication-aware UAV MARL frameworks further incorporate distance-limited communication graphs, neighbor message aggregation, and attention mechanisms to improve decentralized coordination under partial observability [4]. Here, communication-aware MARL refers to the learning of cooperative policies while explicitly accounting for communication conditions, such as which agents can exchange information, how communication links evolve with agent mobility, and how messages from neighboring agents are aggregated. For UAV swarms, this is commonly modeled by a dynamic communication graph, where two UAVs can exchange messages only when their distance is within a communication radius. This setting is more realistic than assuming unrestricted global communication, but it still leaves open the question of whether the transmitted messages are compact, task-relevant, and non-redundant.

Despite these advances, communication remains a major bottleneck for the deployment of MARL-based UAV swarms in realistic environments. UAV-to-UAV communication is constrained by limited bandwidth, intermittent connectivity, energy consumption, communication range, latency, and packet loss. Existing communication learning methods in MARL have explored several directions. Early works studied differentiable and continuous message passing, enabling agents to exchange learnable hidden representations through end-to-end training [14,15]. Subsequent studies introduced attentional and targeted communication mechanisms, allowing agents to selectively decide when to communicate and which agents should receive messages [16,17,18]. Communication scheduling has also been investigated to reduce unnecessary message exchange under limited communication resources [19]. More recently, information-theoretic and graph-based communication methods have been developed to improve message efficiency and robustness in cooperative MARL [20,21,22]. These methods have significantly improved cooperative decision-making by learning when, with whom, and how agents should communicate. However, many of them mainly focus on communication topology, message routing, or aggregation mechanisms, while the information content of transmitted messages is often only implicitly regularized.

This limitation is particularly important in UAV swarms. When multiple UAVs observe overlapping regions or similar environmental cues, dense latent messages may contain substantial redundant or task-irrelevant information. Transmitting such messages not only wastes bandwidth and energy, but may also degrade coordination by introducing noisy or duplicated information into the decision process. Therefore, a key question remains insufficiently explored: what is the minimal amount of information that each UAV should transmit in order to support effective cooperative decision-making?

The information bottleneck (IB) principle provides a principled answer to this question. Originally proposed for the learning of compressed yet predictive representations, IB seeks representations that discard irrelevant information from the input while preserving information useful for the target variable [23,24]. This idea has been extended to reinforcement learning and multi-agent systems, where compact representations can improve sample efficiency, robustness, and scalability [25,26,27]. In the context of communication learning, IB has also been used to learn low-entropy and informative messages under limited bandwidth constraints [20]. Nevertheless, existing IB-based MARL methods are usually developed for generic multi-agent benchmarks and do not explicitly address the practical constraints of UAV swarm coordination, such as distance-limited communication graphs, decentralized execution, dynamic neighbor sets, and redundancy among spatially distributed UAV messages.

In this paper, we propose an information bottleneck framework for communication-efficient multi-agent reinforcement learning in UAV swarms. Our central idea is to formulate inter-UAV communication as a minimally sufficient message-learning problem. Instead of transmitting dense latent features, each UAV encodes its local observation into a compact stochastic message that is regularized to preserve task-relevant information while discarding irrelevant or redundant information. The compressed messages are then exchanged over a distance-limited communication graph and aggregated by neighboring UAVs for decentralized policy execution.

The proposed framework, termed IB-CEMARL, integrates information-theoretic message regularization into a communication-aware MARL architecture. Specifically, each UAV first extracts a local hidden representation from its partial observation, then generates a compact stochastic message through a reparameterized message encoder. A Cauchy–Schwarz (CS) divergence-based compression objective [28,29] discourages the message from retaining excessive local information, while a task-relevance objective preserves information useful for cooperative decision-making. To further improve communication efficiency in dense swarm configurations, we introduce a redundancy-aware regularization term that discourages neighboring UAVs from transmitting statistically duplicated information.

The main contributions of this work are summarized as follows:

- We formulate communication-efficient UAV swarm coordination as a minimally sufficient message-learning problem from an information-theoretic perspective. Unlike existing communication-aware MARL methods that mainly optimize communication topology or message aggregation, our formulation explicitly considers what information should be transmitted under bandwidth, connectivity, and redundancy constraints.
- We propose IB-CEMARL, an information-bottleneck-guided communication framework that learns compact, task-relevant, and non-redundant inter-UAV messages. In contrast with existing IB-based communication methods that mainly focus on message compression, our framework explicitly models inter-agent message redundancy. Cauchy–Schwarz divergence-based quadratic mutual information is used as a unified dependence measure to jointly optimize message compression, task-relevance preservation, and redundancy reduction.
- We extensively evaluate IB-CEMARL under realistic communication constraints, including packet loss, limited communication ranges, varying swarm sizes, and message dimensions. The results demonstrate that the proposed framework achieves competitive cooperative performance while substantially reducing message dependence and improving robustness and generalization compared with representative communication-aware MARL baselines.

## 2. Related Work

### 2.1. Multi-Agent Reinforcement Learning for UAV Swarms

Multi-agent reinforcement learning (MARL) has been widely studied as a learning-based framework for cooperative decision-making among multiple autonomous agents. Compared with single-agent reinforcement learning, MARL is particularly suitable for UAV swarm coordination because each UAV must make local decisions while interacting with other UAVs and sharing a common mission objective. Typical UAV swarm tasks, such as cooperative sensing, area coverage, communication relay deployment, search and rescue, and surveillance, naturally involve partial observability, decentralized execution, dynamic interactions, and long-horizon coordination [3,5,30].

A common paradigm in cooperative MARL is centralized training with decentralized execution (CTDE). During training, agents may access global state information or centralized critics, while during execution, each agent selects actions based only on its local observation and available messages. This paradigm has been adopted in many representative MARL algorithms, including multi-agent actor–critic methods, value decomposition methods, counterfactual policy gradients, and proximal policy optimization variants [6,7,8,13,31]. For UAV swarms, CTDE is especially attractive because decentralized execution avoids reliance on a central controller and better matches practical aerial networks, where global communication may be unavailable or unreliable.

Recent UAV-oriented MARL studies have further explored swarm deployment, cooperative coverage, ground surveillance, collision avoidance, and UAV-aided communication or edge-computing tasks [4,10,11,12]. In these scenarios, UAVs must coordinate their movements and sensing behaviors under changing spatial configurations and limited local observations. Communication among UAVs can significantly improve coordination by allowing each agent to infer the states, intentions, or observations of nearby agents. However, this also introduces an important practical challenge: the learning framework must not only optimize cooperative control policies but also account for the limited and dynamic communication capability of UAV swarms.

### 2.2. Information Bottleneck for Representation Learning in Reinforcement Learning

The information bottleneck (IB) principle provides a principled framework for learning compact yet task-relevant representations by compressing input variables while preserving information useful for prediction or decision-making [23,24]. Given an input variable (*X*), a learned representation (*Z*), and a task-relevant variable (*Y*), the IB objective can be written as

(1) maxI(Z;Y)−βI(X;Z),

where I(X;Z) penalizes the amount of input information retained in the representation, I(Z;Y) encourages the representation to preserve task-relevant information, and β controls the trade-off between compression and prediction. This formulation naturally matches reinforcement learning, where agents should retain information useful for action selection and long-term return while discarding nuisance or task-irrelevant factors.

Beyond variational IB formulations [24], non-parametric information-theoretic approaches have also been explored for representation learning. Matrix-based entropy functionals provide a kernel-based framework for estimating entropy, mutual information, and statistical dependence without explicitly modeling the underlying probability distributions [32]. These approaches have been applied to deterministic information bottleneck learning and dependence-based representation optimization [33]. Compared with variational IB methods, matrix-based entropy formulations avoid explicit prior assumptions and provide flexible non-parametric estimators. However, existing matrix-based IB methods mainly focus on single-agent representation learning and have not considered communication-efficient multi-agent reinforcement learning, where messages must be simultaneously compressed, task-relevant, and non-redundant under dynamic communication constraints.

In reinforcement learning, IB-based representation learning has been used to encourage agents to discard nuisance information, improve exploration, enhance robustness, and learn more generalizable state abstractions [25,26,34,35]. Compared with its use in supervised learning and single-agent reinforcement learning, IB remains less explored in multi-agent reinforcement learning. A few recent studies have investigated IB regularization for efficient inter-agent messages, compact behavior representations, and robust graph-based message passing [20,21,27]. These studies suggest that information-theoretic compression can help reduce redundant or irrelevant information in multi-agent decision-making.

However, most existing IB-based MARL methods are developed for generic multi-agent benchmarks and do not explicitly address UAV-specific communication constraints, such as distance-limited connectivity, dynamic neighbor sets, bandwidth limitations, and spatial redundancy among neighboring UAVs. Our work extends IB-based representation learning to communication-efficient UAV swarm coordination, where each UAV learns to transmit minimally sufficient messages for decentralized cooperative control.

### 2.3. Communication-Efficient UAV Swarm Coordination

Communication is a critical component of UAV swarm coordination, but it is also one of the main bottlenecks in realistic deployment. Unlike idealized MARL settings where agents may freely access global information or exchange dense hidden states, UAV-to-UAV communication is constrained by bandwidth, energy consumption, communication range, latency, interference, and packet loss. These constraints are especially significant in disaster response, wildfire monitoring, remote-area surveillance, and aerial relay deployment, where UAVs may operate in infrastructure-limited environments and must rely on peer-to-peer communication [3,5,30].

To address these challenges, recent studies have investigated communication-aware coordination for UAV swarms. In such settings, communication-aware MARL usually refers to the learning of cooperative policies while explicitly considering communication conditions, such as which UAVs can exchange information, how the communication graph changes as UAVs move, and how messages from neighboring UAVs are aggregated. For example, distance-limited communication graphs provide a more realistic model than unrestricted global communication, since two UAVs can exchange messages only when they are within a feasible communication radius. Attention-based or graph-based aggregation mechanisms can then be used to combine information from dynamically changing neighbors [4,36,37].

Although these methods improve decentralized coordination under communication constraints, most existing UAV-oriented MARL frameworks mainly focus on communication topology, message routing, neighbor selection, or message aggregation. The information content of the transmitted messages is often only implicitly controlled by the downstream task loss. As a result, UAVs may still transmit dense latent features containing redundant, noisy, or task-irrelevant information. This is inefficient in UAV swarms because neighboring UAVs often observe overlapping regions or similar environmental cues, leading to duplicated information exchange and unnecessary communication overhead.

Communication-efficient UAV swarm coordination therefore requires a more explicit treatment of what information should be transmitted. Instead of only asking which UAVs can communicate or how messages should be aggregated, one should also ask how much information each UAV should transmit and whether the transmitted information is useful for cooperative decision-making. This motivates our information bottleneck formulation, where inter-UAV communication is modeled as a minimally sufficient message-learning problem. In contrast to prior communication-aware UAV MARL methods, our approach explicitly regularizes the information content of transmitted messages, encouraging each UAV to send compact, task-relevant, and non-redundant information for decentralized cooperative control.

## 3. Method

**Figure 1:**
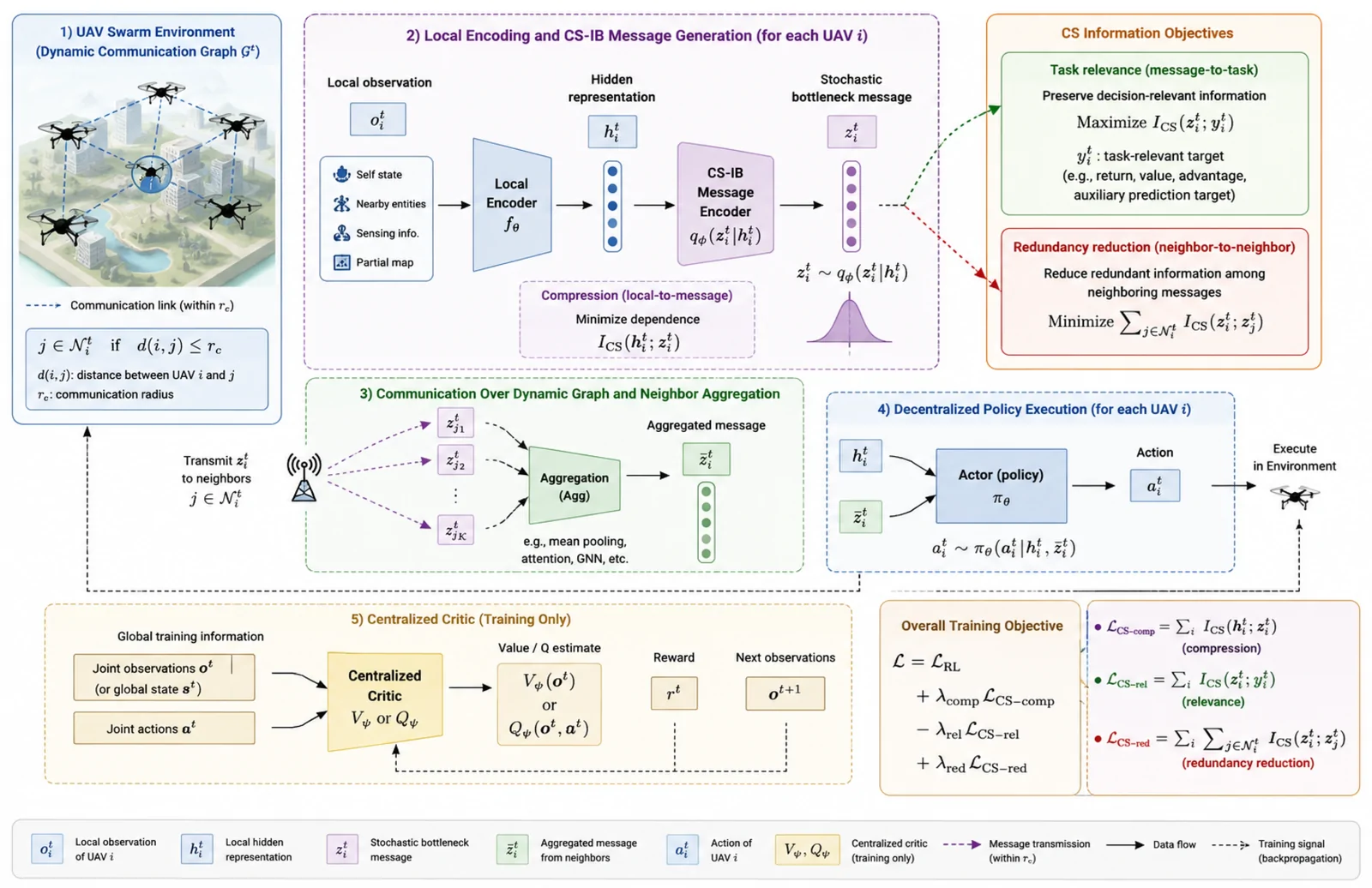
Overview of the proposed IB-CEMARL framework. Each UAV observes only local environmental and agent information, which is encoded into a hidden representation and further compressed into a stochastic bottleneck message. The transmitted messages are exchanged over a dynamic distance-limited communication graph and aggregated by neighboring UAVs for decentralized policy execution. To enable communication-efficient coordination, we instantiate the information bottleneck objective using Cauchy–Schwarz information, which encourages message compression, preserves task-relevant information, and reduces redundancy among neighboring UAV messages. During training, a centralized critic uses global information to optimize the policy under the CTDE paradigm, while during execution, each UAV acts only based on its local observation and received compact messages.

Figure 1 provides an overview of the proposed IB-CEMARL framework. Each UAV first encodes its local observation into a hidden representation, then generates a stochastic bottleneck message for communication with neighboring UAVs over a dynamic distance-limited graph. The information bottleneck objective is instantiated through Cauchy–Schwarz divergence-based quadratic mutual information regularization, which promotes message compression, decision-relevant information preservation, and redundancy reduction among neighboring UAV messages.

### 3.1. Problem Formulation

We consider a cooperative UAV swarm consisting of *N* agents. At each time step (*t*), UAV *i* receives a local observation ($o_{i}^{t}\in O_{i}$), which may include its own state, nearby entities, local sensing information, and partial observations of the environment. Due to limitations in sensing and communication capabilities, each UAV only has access to partial information. The goal of the swarm is to learn decentralized policies that maximize the expected cumulative team reward.

We formulate the problem as a partially observable cooperative multi-agent reinforcement learning problem. Let $o^{t}=\left\{o_{1}^{t},\ldots ,o_{N}^{t}\right\}$ denote the joint observation and $a^{t}=\left\{a_{1}^{t},\ldots ,a_{N}^{t}\right\}$ denote the joint action. The environment returns a shared reward ($r^{t}$) and transitions to the next state according to the system dynamics. The objective is to maximize

(2) $$J\left(\theta \right)=E_{\pi_{\theta }}\left[\sum_{t=0}^{T}\gamma^{t}r^{t}\right],$$

where γ is the discount factor and $\pi_{\theta }$ denotes the learned multi-agent policy.

In realistic UAV swarms, communication is constrained by the spatial configuration of the agents. We define a dynamic communication graph ($G^{t}=\left(V,E^{t}\right)$) where each node corresponds to a UAV and an edge ($\left(i,j\right)\in E^{t}$) exists if UAV *i* and UAV *j* can communicate at time *t*. A common distance-limited communication model is

(3) $$j\in N_{i}^{t}\;\mathrm{if}\;d\left(i,j\right)\leq r_{c},$$

where $N_{i}^{t}$ is the neighbor set of UAV *i*, d(i,j) is the Euclidean distance between two UAVs, and $r_{c}$ is the communication radius. Each UAV can exchange messages only with agents in its current neighborhood. Therefore, the policy of UAV *i* is conditioned on its local observation and the messages received from neighboring UAVs:

(4) $$a_{i}^{t}∼\pi_{\theta }\left(a_{i}^{t}|o_{i}^{t},\left\{m_{j}^{t}:j\in N_{i}^{t}\right\}\right).$$

Our goal is not only to learn effective cooperative policies but also to make the exchanged inter-UAV messages compact, task-relevant, and non-redundant.

### 3.2. Communication-Aware UAV MARL Framework

We adopt a framework of centralized training with decentralized execution. During centralized training, the critic can access global information, such as the joint state, joint observations, or joint actions. During decentralized execution, each UAV selects its action based only on its local observation and the messages received from its communication neighbors.

For each UAV (*i*), a local encoder first maps the observation ($o_{i}^{t}$) to a hidden representation:

(5) $$h_{i}^{t}=f_{\theta }\left(o_{i}^{t}\right).$$

This representation summarizes the local state of the UAV and its nearby environment. In standard communication-aware MARL, each UAV directly transmits a deterministic hidden message derived from $h_{i}^{t}$ to its neighbors. The receiving UAV then aggregates messages from its neighbor set:

(6) $${\bar{m}}_{i}^{t}=\mathrm{Agg}\left(\{m_{j}^{t}:j\in N_{i}^{t}\}\right),$$

where Agg(·) can be implemented by mean pooling, graph aggregation, or attention-based aggregation.

The decentralized actor then selects an action according to

(7) $$a_{i}^{t}∼\pi_{\theta }\left(a_{i}^{t}|h_{i}^{t},{\bar{m}}_{i}^{t}\right).$$

A centralized critic estimates the value function or action-value function using global training information:

(8) $$V_{\psi }\left(o^{t}\right)\;\mathrm{or}\;Q_{\psi }\left(o^{t},a^{t}\right).$$

This architecture captures the communication topology and neighbor message aggregation in UAV swarms. However, if the transmitted message ($m_{i}^{t}$) is simply a dense latent feature, it may contain redundant or task-irrelevant information. To address this issue, we introduce an information bottleneck message encoder.

### 3.3. Information Bottleneck Message Encoding

The central idea of our method is to formulate inter-UAV communication as a minimal sufficient message-learning problem. Instead of transmitting a dense deterministic feature, each UAV encodes its local representation into a compact stochastic message:

(9) $$z_{i}^{t}∼q_{\phi }\left(z_{i}^{t}|h_{i}^{t}\right),$$

where $z_{i}^{t}$ denotes the bottleneck message transmitted by UAV *i*. The message encoder is parameterized as a Gaussian distribution:

(10) $$q_{\phi }\left(z_{i}^{t}|h_{i}^{t}\right)=N\left(\mu_{\phi }\left(h_{i}^{t}\right),\mathrm{diag}\left(\sigma_{\phi }^{2}\left(h_{i}^{t}\right)\right)\right).$$

The message is sampled using the reparameterization trick:

(11) $$z_{i}^{t}=\mu_{\phi }\left(h_{i}^{t}\right)+\sigma_{\phi }\left(h_{i}^{t}\right)⊙\epsilon ,\;\epsilon ∼N\left(0,I\right).$$

Following the information bottleneck principle, the transmitted message should be compact while preserving information that is useful for cooperative decision-making. This objective can be expressed as

(12) $$\max\;I\left(z_{i}^{t};y_{i}^{t}\right)-\beta I\left(h_{i}^{t};z_{i}^{t}\right),$$

where $y_{i}^{t}$ denotes a decision-relevant target, such as a return, advantage estimate, action-related target, or value target. The term $I(h_{i}^{t};z_{i}^{t})$ discourages the message from retaining excessive information from the local representation, whereas $I(z_{i}^{t};y_{i}^{t})$ encourages it to preserve information relevant to policy learning.

A conventional variational implementation typically upper-bounds the compression term using the KL divergence between the conditional message distribution and a predefined prior [24]. Although effective, such a formulation depends on the choice of prior and does not directly provide a unified measure for both local-to-message dependence and inter-agent message redundancy. We therefore instantiate the bottleneck objective using Cauchy–Schwarz divergence-based quadratic mutual information [28], allowing message compression, task relevance, and inter-agent redundancy to be regularized within a unified dependence-measure framework.

### 3.4. Task-Relevance and Redundancy-Aware CS Message Regularization

Only compressing messages is insufficient because overly compressed representations may discard information that is essential for cooperative decision-making. Therefore, an efficient inter-UAV communication mechanism should simultaneously satisfy three requirements: (1) removing irrelevant information from local representations, (2) preserving information useful for task execution, and (3) avoiding duplicated information exchange among neighboring UAVs.

To achieve this goal, we instantiate the information bottleneck objective using Cauchy–Schwarz (CS) information as a unified dependence measure. Compared with conventional variational IB formulations based on KL divergence, CS information directly measures statistical dependence between variables without requiring a predefined variational prior [28]. This property is particularly desirable for UAV swarm communication, where message distributions dynamically change with agent locations, communication topology, and environmental conditions. Moreover, CS information is symmetric and can naturally characterize the dependence among multiple communicated messages, enabling a unified formulation of local-to-message compression, message-task relevance, and inter-agent redundancy reduction within the same objective [38].

Therefore, the advantage of CS information in our framework is not that it universally replaces KL-based IB formulations but that it provides a convenient and consistent dependence measure for simultaneously modeling message compression, task relevance, and inter-agent redundancy.

Given two random variables (*X* and *Z*), the CS information is defined as

(13) $$I_{\mathrm{CS}}\left(X;Z\right)=D_{\mathrm{CS}}\left(p_{XZ},p_{X}p_{Z}\right),$$

where $D_{\mathrm{CS}}\left(\cdot ,\cdot \right)$ denotes the Cauchy–Schwarz divergence between the joint distribution ($p_{XZ}$) and the product of marginals ($p_{X}p_{Z}$). This quantity measures the statistical dependence between *X* and *Z*. If *X* and *Z* are independent, then $p_{XZ}=p_{X}p_{Z}$, and the corresponding dependence is minimized.

In our UAV swarm setting, CS information is used to regularize three aspects of inter-UAV communication. First, we introduce a message compression term, i.e.,

(14) $$L_{\mathrm{CS}-\mathrm{comp}}=\frac{1}{N}\sum_{i=1}^{N}I_{\mathrm{CS}}\left(h_{i}^{t};z_{i}^{t}\right),$$

which discourages the transmitted message ($z_{i}^{t}$) from preserving excessive information from the local representation ($h_{i}^{t}$). This term encourages each UAV to transmit only compact information rather than dense latent features.

Second, to ensure that the compressed messages remain useful for cooperative decision-making, we introduce a task-relevance term. Let $y_{i}^{t}$ denote a decision-relevant target, such as the empirical return, value target, advantage estimate, or an auxiliary action-related prediction target. The CS-based task-relevance objective is defined as

(15) $$L_{\mathrm{CS}-\mathrm{rel}}=-\frac{1}{N}\sum_{i=1}^{N}I_{\mathrm{CS}}\left(z_{i}^{t};y_{i}^{t}\right),$$

where the negative sign indicates that the dependence between the transmitted message and the decision-relevant target is maximized. This term encourages $z_{i}^{t}$ to preserve information that is useful for policy learning and cooperative control.

Third, we introduce a redundancy-aware regularization term to reduce duplicated information among neighboring UAVs. In dense UAV swarms, nearby agents may observe overlapping regions and therefore generate highly similar messages. Such duplicated messages increase communication cost without providing additional benefits for coordination. We define the CS-based redundancy reduction term as

(16) $$L_{\mathrm{CS}-\mathrm{red}}=\frac{1}{|E^{t}|}\sum_{\left(i,j\right)\in E^{t}}I_{\mathrm{CS}}\left(z_{i}^{t};z_{j}^{t}\right),$$

which penalizes statistical dependence among messages transmitted by neighboring UAVs. Minimizing this term encourages neighboring UAVs to transmit complementary rather than duplicated information.

When $E^{t}$ is empty, the redundancy term is set to zero. Averaging over the active communication edges prevents the scale of the regularizer from increasing solely with the swarm size or communication radius.

In practice, the CS information terms are estimated from mini-batch samples using Gaussian KDE. The resulting differentiable estimator is provided in Appendix A.

The resulting message received by UAV *i* is computed by aggregating the bottleneck messages from its communication neighbors:

(17) $${\bar{z}}_{i}^{t}=\mathrm{Agg}\left(\{z_{j}^{t}:j\in N_{i}^{t}\}\right).$$

The decentralized policy is then written as

(18) $$a_{i}^{t}∼\pi_{\theta }\left(a_{i}^{t}|h_{i}^{t},{\bar{z}}_{i}^{t}\right).$$

Thus, each UAV makes decisions based on its own local representation and compact, task-relevant, and non-redundant messages received from nearby UAVs.

### 3.5. Overall Objective and Training

The overall training objective combines the MARL objective with the proposed CS-based information bottleneck regularization terms. For an actor–critic implementation, the reinforcement learning loss can be written as

(19) $$L_{\mathrm{RL}}=L_{\mathrm{actor}}+c_{v}L_{\mathrm{critic}}-c_{e}H\left(\pi_{\theta }\right),$$

where $L_{\mathrm{actor}}$ is the policy optimization loss, $L_{\mathrm{critic}}$ is the value loss, $H(\pi_{\theta })$ is the policy entropy bonus, and $c_{v}$ and $c_{e}$ are weighting coefficients.

The full objective of IB-CEMARL is defined as

(20) $$L=L_{\mathrm{RL}}+\lambda_{\mathrm{comp}}L_{\mathrm{CS}-\mathrm{comp}}+\lambda_{\mathrm{rel}}L_{\mathrm{CS}-\mathrm{rel}}+\lambda_{\mathrm{red}}L_{\mathrm{CS}-\mathrm{red}},$$

where $\lambda_{\mathrm{comp}}$, $\lambda_{\mathrm{rel}}$, and $\lambda_{\mathrm{red}}$ control the strengths of message compression, task relevance, and redundancy reduction, respectively.

During training, trajectories are collected from the UAV swarm under the current decentralized policies. The centralized critic is updated using global training information, while the actor and message encoder are updated using local observations, received bottleneck messages, CS information regularization, and policy gradients. During execution, each UAV only requires its own observation and the compact messages received from neighboring UAVs. No global state or centralized controller is required. Therefore, the proposed framework preserves decentralized execution while explicitly improving communication efficiency through CS-based information-bottleneck message learning. The complete training procedure of IB-CEMARL is summarized in Algorithm 1.
**Algorithm 1** Training Procedure of the Proposed IB-CEMARL**Require:** 
UAV environment E; number of UAVs *N*; communication radius $r_{c}$; actor $\pi_{\theta }$;     critic $V_{\psi }$; local encoder $f_{\theta }$; message encoder $q_{\phi }$; aggregator Agg(·); learning rate η;     weights $\lambda_{\mathrm{comp}}$, $\lambda_{\mathrm{rel}}$, $\lambda_{\mathrm{red}}$  1:**while** not converged **do**  2:      Initialize the UAV swarm and receive local observations ${\left\{o_{i}^{0}\right\}}_{i=1}^{N}$  3:      **for** t=0,1,…,T **do**  4:            **for** each UAV i=1,…,N **do**  5:                 Construct neighbor set $N_{i}^{t}=\left\{j|d\left(i,j\right)\leq r_{c},\;j\neq i\right\}$  6:                 Encode local observation $h_{i}^{t}=f_{\theta }\left(o_{i}^{t}\right)$  7:                 Generate stochastic bottleneck message $z_{i}^{t}∼q_{\phi }\left(z_{i}^{t}|h_{i}^{t}\right)$  8:           **end for**  9:           **for** each UAV i=1,…,N **do**10:                 Aggregate received messages:$${\bar{z}}_{i}^{t}=\mathrm{Agg}\left(\{z_{j}^{t}:j\in N_{i}^{t}\}\right)$$11:                 Sample decentralized action $a_{i}^{t}∼\pi_{\theta }\left(a_{i}^{t}|h_{i}^{t},{\bar{z}}_{i}^{t}\right)$12:           **end for**13:           Execute joint action $a^{t}={\left\{a_{i}^{t}\right\}}_{i=1}^{N}$14:           Observe reward $r^{t}$ and next observations ${\left\{o_{i}^{t+1}\right\}}_{i=1}^{N}$15:           Store transition $\left(o^{t},a^{t},r^{t},o^{t+1},G^{t}\right)$16:     **end for**17:     Estimate decision-relevant targets $\left\{y_{i}^{t}\right\}$, e.g., returns, value targets, or advantages18:     Compute reinforcement learning loss:$$L_{\mathrm{RL}}=L_{\mathrm{actor}}+c_{v}L_{\mathrm{critic}}-c_{e}H\left(\pi_{\theta }\right)$$19:     Compute CS information regularization terms:$$L_{\mathrm{CS}-\mathrm{comp}}=\sum_{t,i}I_{\mathrm{CS}}\left(h_{i}^{t};z_{i}^{t}\right),\;L_{\mathrm{CS}-\mathrm{rel}}=-\sum_{t,i}I_{\mathrm{CS}}\left(z_{i}^{t};y_{i}^{t}\right),$$$$L_{\mathrm{CS}-\mathrm{red}}=\sum_{t,i}\sum_{j\in N_{i}^{t}}I_{\mathrm{CS}}\left(z_{i}^{t};z_{j}^{t}\right)$$20:     Optimize the overall objective:$$L=L_{\mathrm{RL}}+\lambda_{\mathrm{comp}}L_{\mathrm{CS}-\mathrm{comp}}+\lambda_{\mathrm{rel}}L_{\mathrm{CS}-\mathrm{rel}}+\lambda_{\mathrm{red}}L_{\mathrm{CS}-\mathrm{red}}$$21:     Update θ, ϕ, and ψ by gradient descent:$$\theta ,\phi ,\psi \leftarrow \theta ,\phi ,\psi -\eta \nabla_{\theta ,\phi ,\psi }L$$22:**end while**

## 4. Experiments

We evaluate the proposed IB-CEMARL framework on communication-constrained UAV swarm coordination tasks. The experiments are designed to answer the following questions: (1) Can IB-CEMARL improve cooperative performance under distance-limited inter-UAV communication? (2) Can the proposed Cauchy–Schwarz information bottleneck reduce local-to-message dependence and statistical redundancy among neighboring UAV messages? (3) How robust is IB-CEMARL under packet loss, limited communication range, and unseen swarm configurations? (4) What are the individual contributions of message compression, task-relevance preservation, and redundancy reduction?

### 4.1. Experimental Setup

#### 4.1.1. UAV Swarm Coordination Task

We consider a cooperative UAV swarm coordination task in a bounded two-dimensional workspace of size L×L. A swarm consisting of *N* UAVs is deployed to collaboratively cover randomly distributed ground targets. Each UAV has only partial observations of the environment, including nearby targets and neighboring UAVs within its sensing radius ($r_{s}$). Therefore, the observation available to each UAV does not contain global target information.

The objective of the swarm is to maximize the overall coverage ratio while maintaining decentralized coordination. A target is considered covered if it is located within the sensing range of at least one UAV. During execution, each UAV selects actions using only its local observation and received messages from communication neighbors.

At each time step (*t*), UAV *i* observes only local information, including its own position, velocity, nearby targets, and neighboring UAVs within its sensing range. The global state is available only during centralized training for critic learning. During execution, each UAV selects actions using its local observation and the compact messages received from communication neighbors.

#### 4.1.2. Communication Model

We use a distance-limited inter-UAV communication model. At time step *t*, UAV *j* belongs to the communication neighborhood of UAV *i* if their Euclidean distance is smaller than a predefined communication radius ($r_{c}$):

(21) $$N_{i}^{t}=\left\{j|d\left(i,j\right)\leq r_{c},\;j\neq i\right\}.$$

The communication radius is intentionally smaller than the workspace size, resulting in a sparse and dynamically changing communication graph rather than global communication. The corresponding dynamic communication graph is denoted as $G^{t}=\left(V,E^{t}\right)$, where each node represents a UAV and each edge represents an available communication link. Messages are exchanged only along the edges of $G^{t}$. To evaluate robustness to unreliable communication, we additionally consider random packet loss, where each transmitted message is dropped with a probability of $p_{\mathrm{loss}}$.

#### 4.1.3. Observation and Action Spaces

The local observation of each UAV consists of its own state and local environmental information:

(22) $$o_{i}^{t}=\left[p_{i}^{t},v_{i}^{t},T_{i}^{t},U_{i}^{t}\right],$$

where $p_{i}^{t}$ and $v_{i}^{t}$ denote the position and velocity of UAV *i*, $T_{i}^{t}$ denotes the locally observed target information, and $U_{i}^{t}$ denotes the locally observed neighboring UAV information. The action of UAV *i* is a continuous movement command, i.e.,

(23) $$a_{i}^{t}=\left[\Delta x_{i}^{t},\Delta y_{i}^{t}\right],$$

which determines the displacement or velocity command of the UAV at the next time step. For three-dimensional deployment tasks, the action can be extended to $a_{i}^{t}=\left[\Delta x_{i}^{t},\Delta y_{i}^{t},\Delta z_{i}^{t}\right]$.

#### 4.1.4. Reward Design

The reward encourages UAVs to maximize cooperative coverage while avoiding inefficient movement and redundant deployment. The shared team reward at time step *t* is defined as

(24) $$r^{t}=\alpha_{\mathrm{cov}}R_{\mathrm{cov}}^{t}-\alpha_{\mathrm{move}}R_{\mathrm{move}}^{t}-\alpha_{\mathrm{col}}R_{\mathrm{col}}^{t},$$

where $R_{\mathrm{cov}}^{t}$ measures the coverage ratio or the number of newly covered targets, $R_{\mathrm{move}}^{t}$ penalizes excessive movement or energy consumption, and $R_{\mathrm{col}}^{t}$ penalizes collisions or unsafe distances between UAVs. The $\alpha_{\mathrm{cov}}$, $\alpha_{\mathrm{move}}$, and $\alpha_{\mathrm{col}}$ coefficients control the relative importance of these terms.

#### 4.1.5. Implementation Details

All methods are implemented using multi-agent proximal policy optimization (MAPPO) [8] as the underlying MARL optimizer. The actor and critic are parameterized by two fully connected hidden layers with 128 units per layer and ReLU activations. The local representation dimension is set to 128, and the default bottleneck message dimension is set to 16. All models are trained for $2\times 10^{6}$ environment steps using the Adam optimizer with a learning rate of $3\times 10^{-4}$, a discount factor of γ=0.99, and a mini-batch size of 256. The PPO clipping coefficient is set to 0.2, the generalized advantage estimation parameter is set to $\lambda_{\mathrm{GAE}}=0.95$, and each policy update uses 10 optimization epochs.

Unless otherwise stated, the environment contains five UAVs and 20 ground targets randomly initialized in a 500×500 workspace. The sensing radius is set to $r_{s}=50$, and the communication radius is set to $r_{c}=100$. Under this configuration, UAVs only perceive local environmental information and can exchange messages with nearby agents within the communication radius, resulting in a sparse and dynamically changing communication graph.

For IB-CEMARL, the regularization coefficients are set to $\lambda_{\mathrm{comp}}=0.01$, $\lambda_{\mathrm{rel}}=0.05$, and $\lambda_{\mathrm{red}}=0.01$. All reported results are averaged over five independent random seeds.

#### 4.1.6. Evaluation Metrics

We evaluate each method using task-performance, communication-efficiency, robustness, and generalization metrics:

- Average return: The discounted cumulative team reward obtained over an evaluation episode;
- Coverage ratio: The proportion of ground targets or areas of interest covered by the UAV swarm, i.e.,
  (25) $$\mathrm{Coverage}=\frac{\mathrm{number}\;\mathrm{of}\;\mathrm{covered}\;\mathrm{targets}}{\mathrm{total}\;\mathrm{number}\;\mathrm{of}\;\mathrm{targets}};$$
- Local-to-message dependence: The average statistical dependence between the local hidden representation and the transmitted message over an episode, i.e.,
  (26) $$D_{\mathrm{local}}=\frac{1}{TN}\sum_{t=1}^{T}\sum_{i=1}^{N}I_{\mathrm{CS}}\left(h_{i}^{t};z_{i}^{t}\right),$$
  where *T* is the episode length and *N* is the number of UAVs. A lower value indicates that the transmitted messages retain less information from the local representations and are therefore more strongly compressed.
- Inter-message dependence: The average statistical dependence among messages transmitted by neighboring UAVs, i.e.,
  (27) $$D_{\mathrm{inter}}=\frac{\sum_{t=1}^{T}\sum_{\left(i,j\right)\in E^{t}}I_{\mathrm{CS}}\left(z_{i}^{t};z_{j}^{t}\right)}{\sum_{t=1}^{T}\left|E^{t}\right|},$$
  where $E^{t}$ denotes the set of active communication edges at time step *t*. A lower value indicates less statistical duplication among neighboring UAV messages. If no communication edge is active during an episode, $D_{\mathrm{inter}}$ is set to zero.
- Robustness and generalization: Robustness is evaluated under different packet-loss rates and communication radii. Generalization is assessed by testing the learned policies on swarm sizes not observed during training.

### 4.2. Baselines

We compare IB-CEMARL with representative non-communication, full-communication, communication-aware, graph-based, and information-bottleneck-based MARL baselines:

- NoComm-MARL: This baseline removes inter-UAV communication. Each UAV selects actions using only its local observation. It is used to evaluate the contribution of inter-agent communication to cooperative UAV coordination.
- FullComm-MARL: This baseline allows every UAV to exchange dense latent features with all other UAVs. It serves as an unrestricted communication reference, although it does not account for communication-range constraints or message redundancy.
- DistComm-MARL: This baseline uses the same distance-limited communication graph as IB-CEMARL, but each UAV transmits a deterministic dense message without information-bottleneck regularization. The implementation follows a recent communication-aware UAV MARL framework with dynamic neighbor communication and message aggregation [4]. It is used to isolate the effect of explicitly regularizing the information content of transmitted messages.
- CommNet: CommNet enables agents to exchange continuous hidden representations through differentiable communication [14]. It serves as a classical continuous communication baseline for cooperative MARL.
- TarMAC: TarMAC learns targeted inter-agent communication through an attention-based mechanism [18]. It allows each agent to selectively aggregate messages from other agents.
- SchedNet: SchedNet learns a communication schedule under limited communication resources [19]. It is included to compare IB-CEMARL with methods that improve communication efficiency by selecting which agents are allowed to transmit.
- GraphComm-MARL: This baseline performs message passing over the dynamic communication graph and aggregates messages only from neighboring UAVs using a graph-based communication module [37]. It represents graph-structured communication-aware MARL without explicit information-bottleneck regularization.
- KL-IB-MARL: This baseline uses the same stochastic message encoder and MARL backbone as IB-CEMARL but adopts a conventional KL-based variational bottleneck [20,24]. Specifically, the CS-based compression term is replaced by
  (28) $$L_{\mathrm{KL}}=\frac{1}{N}\sum_{i=1}^{N}D_{\mathrm{KL}}\left(q_{\phi }\left(z_{i}^{t}|h_{i}^{t}\right)\;∥\;p\left(z_{i}\right)\right),$$
  where $p(z_{i})$ is a standard Gaussian prior. The same task-relevance mechanism is retained, while the CS-based inter-message redundancy regularizer is removed. This comparison isolates the effect of the proposed CS-based dependence regularization.
- KL-IB-Red: To fairly compare the dependence measure used for redundancy reduction, this variant augments KL-IB-MARL with a symmetric KL-based inter-agent redundancy penalty, i.e.,
  $$L_{KL-red}=\frac{1}{|E^{t}|}\sum_{\left(i,j\right)\in E^{t}}D_{SKL}\left(q_{\phi }\left(z_{i}|h_{i}\right),q_{\phi }\left(z_{j}|h_{j}\right)\right).$$
  All other components are identical to IB-CEMARL.
- IB-CEMARL: This is the proposed method. Each UAV generates a stochastic bottleneck message and employs Cauchy–Schwarz information regularization to jointly promote message compression, task-relevant information preservation, and redundancy reduction among neighboring UAV messages.

Table 1 summarizes the characteristics of all baselines.

### 4.3. Main Performance Comparison

We first compare IB-CEMARL with representative MARL baselines under the standard communication-constrained UAV swarm setting. The results are reported in Table 2. We evaluate both cooperative task performance and the information characteristics of the learned communication, including the average return, coverage ratio, local-to-message dependence, and inter-message dependence.

As shown in Table 2, communication generally improves cooperative performance compared with the no-communication baseline. However, dense communication methods retain relatively high dependence between local representations and transmitted messages and also exhibit considerable statistical dependence among neighboring messages. KL-IB-MARL reduces the local-to-message dependence through variational bottleneck regularization. IB-CEMARL achieves the lowest local-message and inter-message dependence while obtaining the highest average return and coverage ratio, suggesting that the proposed CS-based regularization learns compact, task-relevant, and less duplicated communication representations.

### 4.4. Ablation Study

We conduct ablation studies to analyze the contribution of each component in IB-CEMARL. Specifically, we evaluate the effects of message compression, task-relevance preservation, and inter-agent redundancy reduction. We compare the full model with the following variants: (1) w/o compression, which removes the CS-based message compression term; (2) w/o task relevance, which removes the task-relevance preservation term; (3) w/o redundancy, which removes the redundancy reduction term among neighboring UAV messages; (4) KL-IB-MARL, which replaces the CS-based information regularization with a conventional KL-based variational bottleneck while removing the redundancy reduction term; (5) KL-IB-Red, which further incorporates a symmetric KL-based inter-agent redundancy penalty while keeping the same message encoder and task-relevance objective as KL-IB-MARL.

The KL-IB-Red variant is introduced to disentangle the effect of explicit redundancy modeling from the choice of dependence measure. Specifically, this comparison evaluates whether the performance improvement originates from reducing inter-agent message redundancy or from the use of CS information as the dependence measure.

Table 3 demonstrates the contribution of each component in IB-CEMARL. Removing the compression term substantially increases the local-to-message dependence, indicating that explicit information compression is necessary to prevent UAVs from transmitting overly detailed latent features.

Removing the task-relevance term results in the largest performance degradation, suggesting that compressed messages alone are insufficient for cooperative decision-making. Without preserving task-relevant information, the learned messages may discard useful information required for effective policy learning.

Removing the redundancy reduction term maintains reasonable task performance but leads to increased inter-message dependence. This verifies that explicitly modeling statistical redundancy among neighboring UAV messages helps reduce duplicated information exchange, particularly when UAVs observe overlapping regions.

The comparison between KL-IB-MARL and KL-IB-Red further confirms the importance of redundancy-aware communication learning. Adding a KL-based redundancy penalty improves both cooperative performance and message efficiency, demonstrating that explicit inter-agent redundancy modeling is beneficial for communication-efficient UAV coordination.

Finally, IB-CEMARL achieves the best overall performance and the lowest message dependence among all variants. This suggests that CS information provides a consistent and unified dependence measure for joint optimization of message compression, task relevance, and redundancy reduction within the proposed information bottleneck framework.

### 4.5. Robustness Analysis

Realistic UAV communication is often unreliable due to packet loss, limited communication range, interference, and changing swarm topology. We therefore evaluate the robustness of IB-CEMARL under three challenging settings: packet loss, varying communication radius, and unseen swarm sizes.

#### 4.5.1. Robustness to Packet Loss

We first evaluate the learned policies under different packet-loss probabilities. During testing, each transmitted message is independently dropped with a probability of $p_{\mathrm{loss}}$. The results are shown in Table 4.

IB-CEMARL degrades more slowly as the packet loss rate increases. The slower performance degradation suggests that the policy relies on more compact and task-relevant communication patterns and is therefore less sensitive to missing messages.

#### 4.5.2. Robustness to Communication Radius

We further evaluate the effect of the communication radius ($r_{c}$). A smaller communication radius results in a sparser communication graph, while a larger radius increases the number of neighbors and may introduce redundant messages. The results are reported in Table 5.

When the communication radius is small, all methods experience performance degradation due to limited neighbor information. However, IB-CEMARL still achieves the best return, showing that compact task-relevant messages are useful, even under sparse communication. When the communication radius becomes larger, dense communication baselines do not improve substantially because additional neighbors may provide redundant information. By contrast, the redundancy-aware CS regularization in IB-CEMARL helps suppress duplicated information and maintains stable performance.

#### 4.5.3. Generalization to Unseen Swarm Sizes

To evaluate scalability, we train all methods with a fixed number of UAVs and test them with unseen swarm sizes. This setting examines whether the learned communication policy can generalize to different numbers of agents. The results are shown in Table 6.

IB-CEMARL generalizes better to unseen swarm sizes. This is because the proposed message regularization learns local and compact communication patterns rather than relying on a fixed global communication structure. The redundancy reduction term is particularly helpful when the number of UAVs increases, since larger swarms are more likely to contain overlapping observations and duplicated messages.

### 4.6. Computational Complexity and Communication Overhead

Although the proposed IB-CEMARL improves communication efficiency by learning compact and non-redundant messages, the additional CS information estimation introduces extra computational cost during training. Therefore, we further evaluate the computational overhead of the proposed framework in terms of training time, GPU memory consumption, and inference speed.

All experiments are conducted using the same hardware and implementation settings as described in Section 4.1.5. We compare IB-CEMARL with the standard MAPPO backbone and KL-IB-MARL, which uses the same stochastic message encoder but adopts a conventional variational bottleneck based on KL divergence. For a fair comparison, all methods use the same actor–critic architecture, batch size, and training steps.

The computational comparison is summarized in Table 7. The results show that IB-CEMARL introduces additional training overhead due to the estimation of CS information terms. This overhead mainly comes from the KDE calculation used to estimate the dependence between local representations, messages, task targets, and neighboring UAV messages. However, the additional cost remains moderate compared with the overall MARL optimization process.

Importantly, the CS information estimation is only required during training. During decentralized execution, each UAV only performs local encoding, message transmission, aggregation, and policy inference. Therefore, IB-CEMARL does not introduce additional inference-time dependence estimation or centralized computation, making it suitable for practical UAV swarm deployment.

Furthermore, although IB-CEMARL requires a slightly higher training cost, it provides more efficient communication representations. The learned messages have lower local-to-message dependence and inter-message redundancy, as demonstrated in Section 4.3. This trade-off is desirable in communication-constrained UAV scenarios, where training-time computation can be exchanged for reduced communication overhead and improved robustness during deployment.

### 4.7. Communication Cost Analysis

Although IB-CEMARL reduces statistical redundancy among exchanged messages, we further analyze its practical communication cost. The communication cost is measured by the number of transmitted message bits. For a message vector with dimension $d_{z}$ and *b* bits per element, the communication cost of each message transmission is

(29) $$C_{\mathrm{msg}}=d_{z}b.$$

We evaluate the trade-off between communication cost and cooperative performance by varying the bottleneck message dimension ($d_{z}$). A smaller message dimension reduces the number of transmitted bits but may discard information necessary for coordination. Therefore, an effective communication framework should achieve a favorable balance between message compactness and task performance.

**Figure 2:**
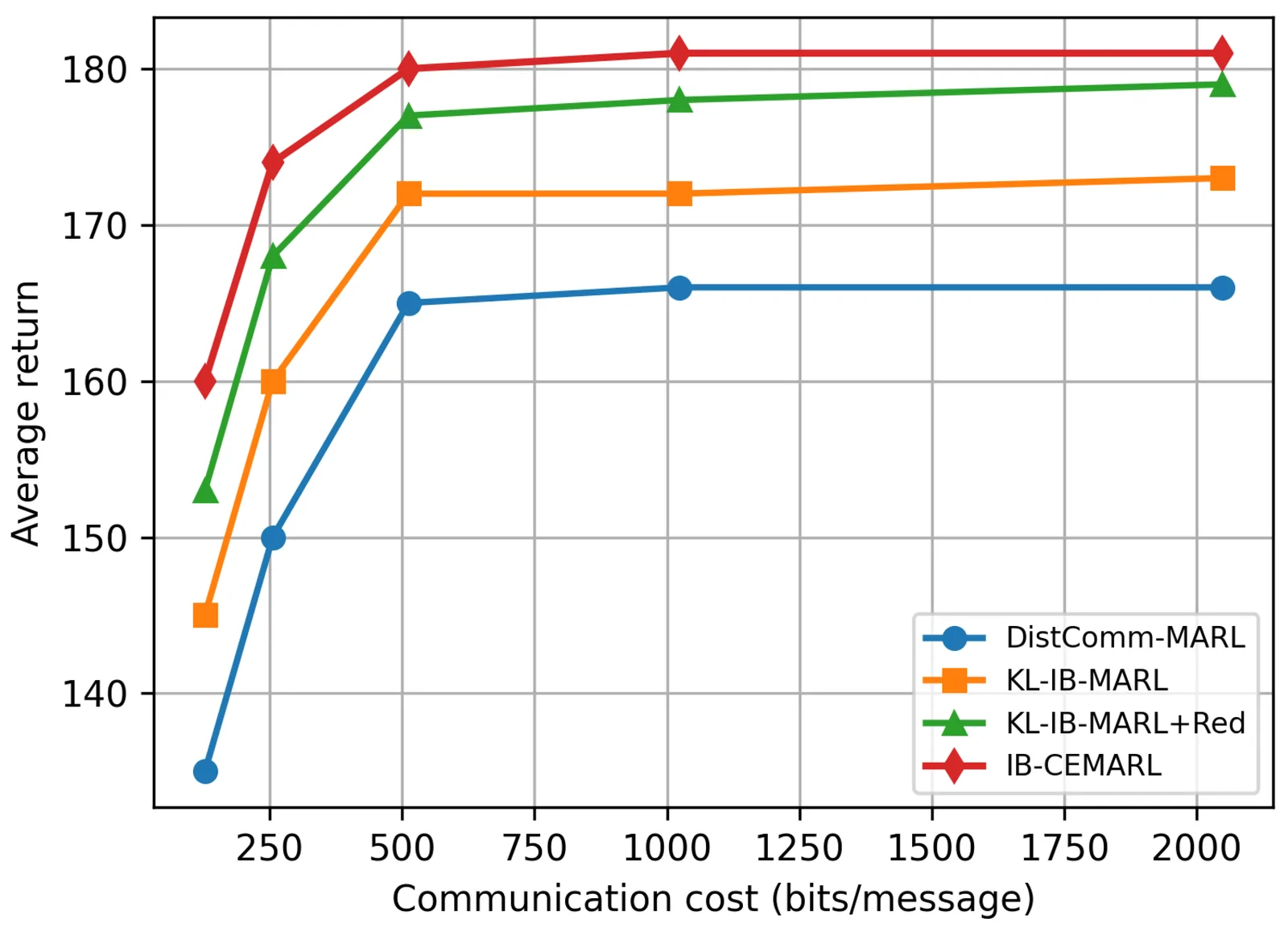
Communication cost–performance trade-off under different message dimensions. The x-axis represents the number of transmitted bits per message, and the y-axis denotes the average cooperative return. IB-CEMARL achieves competitive performance with fewer transmitted bits by learning compact, task-relevant, and non-redundant communication messages.

As shown in Figure 2, IB-CEMARL maintains competitive cooperative performance with smaller message dimensions compared with communication-aware MARL baselines. This indicates that the proposed information-bottleneck objective enables UAVs to exchange compact and task-relevant messages rather than relying on unnecessarily high-dimensional latent features. Furthermore, the results demonstrate that reducing statistical redundancy among messages translates into improved communication efficiency under limited message budgets.

### 4.8. Communication Efficiency and Message Analysis

Since the goal of IB-CEMARL is communication-efficient coordination, we further analyze the learned messages. We evaluate the trade-off between cooperative performance and communication cost and examine whether the proposed redundancy reduction term indeed decreases dependence among neighboring UAV messages.

#### 4.8.1. Performance Under Different Transmitted Message Dimensions

We vary the transmitted message dimension ($d_{z}$) to evaluate how effectively each method uses a fixed-dimensional communication channel. The results are shown in Table 8.

IB-CEMARL achieves stronger performance under small message dimensions, indicating that the learned messages are more compact and informative. When the message dimension increases, the performance gain of dense-message baselines saturates, while IB-CEMARL maintains a better trade-off between communication cost and cooperative return. This supports the effectiveness of learning minimally sufficient messages for UAV swarm coordination.

#### 4.8.2. Message Redundancy Analysis

We also compute the average message redundancy among neighboring UAVs using CS information. Lower message dependence indicates less statistical duplication among neighboring messages. When considered together with the maintained task performance, this suggests that neighboring UAVs learn to communicate more complementary task-relevant information. The results are shown in Table 9.

The redundancy of IB-CEMARL decreases consistently during training, showing that the CS-based redundancy term effectively discourages duplicated messages among neighboring UAVs. This effect is important in dense swarm scenarios, where nearby UAVs often observe overlapping targets or similar environmental cues. By reducing message dependence, IB-CEMARL encourages complementary communication and improves communication efficiency.

### 4.9. Visualization

**Figure 3:**
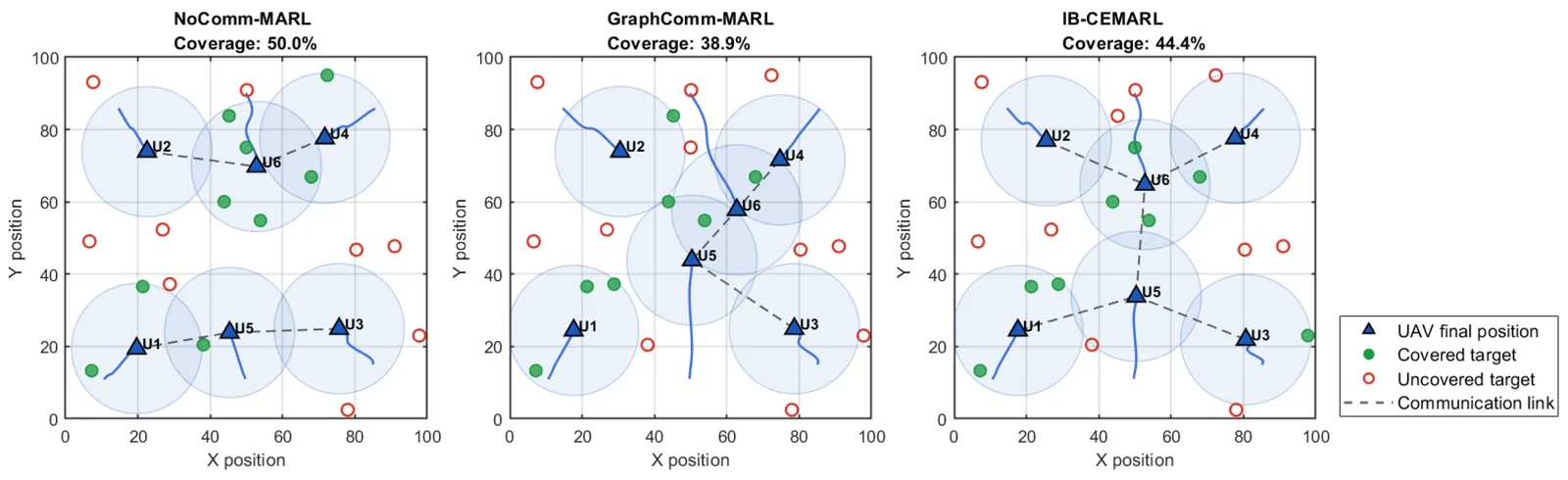
Visualization of UAV trajectories, target coverage, and communication links under different methods. Compared with the no-communication and graph-based communication baselines, IB-CEMARL produces more balanced UAV deployment and improves target coverage while maintaining compact distance-limited inter-UAV communication.

Finally, we visualize representative UAV trajectories, coverage patterns, communication links, and message redundancy. Figure 3 shows the final deployment and trajectories of different methods. In the no-communication baseline, UAVs tend to form locally suboptimal deployment patterns because each agent only has access to its own observation. Graph-based communication improves coordination by allowing UAVs to exchange information with nearby agents, but it may still produce overlapping coverage regions and redundant communication links. In contrast, IB-CEMARL leads to more balanced UAV deployment and more compact inter-UAV communication, resulting in improved target coverage under the same distance-limited communication setting.

**Figure 4:**
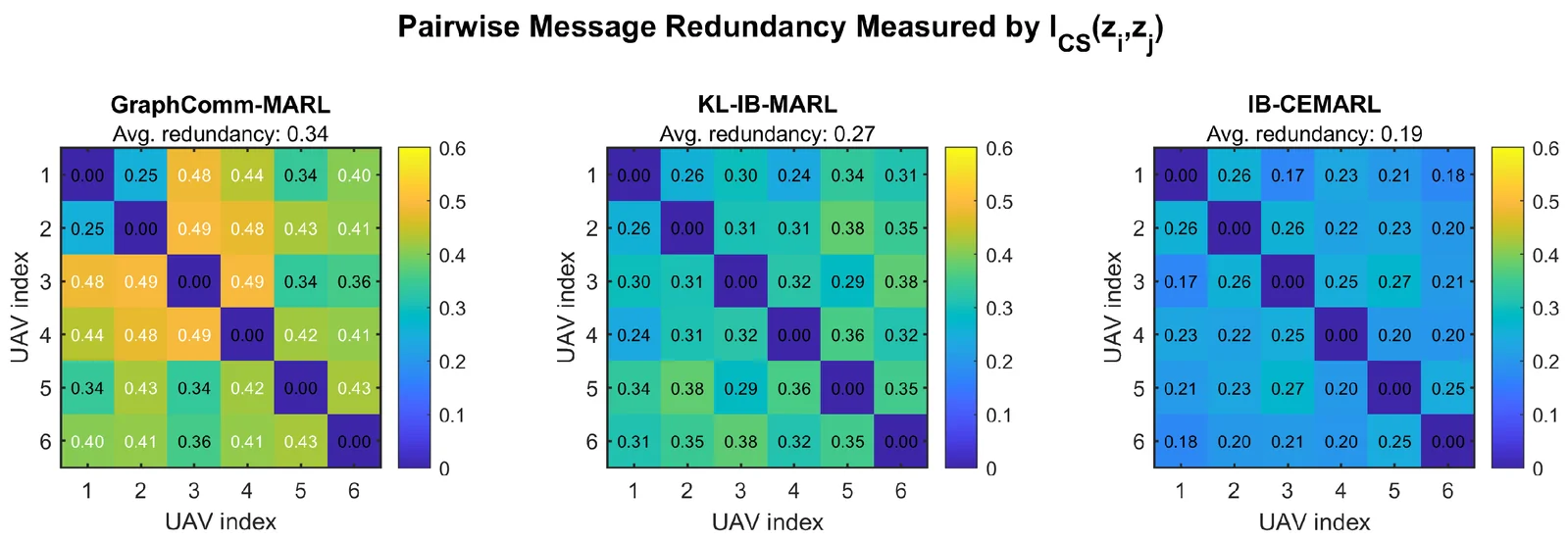
Pairwise message redundancy measured by $I_{\mathrm{CS}}\left(z_{i}^{t},z_{j}^{t}\right)$. Lower off-diagonal values indicate less duplicated information among UAV messages. IB-CEMARL produces lower inter-agent message dependence than graph-based dense communication and KL-based bottleneck communication, demonstrating the effectiveness of the proposed redundancy-aware CS regularization.

To further examine the learned messages, Figure 4 visualizes the pairwise message redundancy matrix, where each entry corresponds to $I_{\mathrm{CS}}\left(z_{i}^{t};z_{j}^{t}\right)$ between two UAVs. Compared with graph-based dense communication and KL-based bottleneck communication, IB-CEMARL produces lower off-diagonal dependence among neighboring UAV messages. This indicates that the redundancy-aware CS regularization effectively suppresses duplicated information and encourages UAVs to transmit more complementary messages for cooperative decision-making.

## 5. Conclusions

This paper presented IB-CEMARL, an information-theoretic framework for the learning of compact and task-relevant communication in cooperative UAV swarms. Inter-UAV communication was formulated as a minimally sufficient message-learning problem in which stochastic bottleneck messages are regularized using Cauchy–Schwarz divergence-based quadratic mutual information. The resulting objective jointly discourages excessive local-to-message dependence, preserves decision-relevant information, and reduces statistical redundancy among neighboring UAV messages.

Experiments on communication-constrained coverage tasks showed that IB-CEMARL improves cooperative return and target coverage while reducing inter-agent message redundancy. The proposed method also exhibited slower performance degradation under packet loss and limited communication range and generalized more effectively to swarm sizes not observed during training. The ablation results further demonstrated that message compression, task relevance, and redundancy reduction make complementary contributions to the final performance.

The present study is limited to a simplified two-dimensional coordination environment with distance-based communication and point-level UAV motion. Consequently, the results should be interpreted as evidence for the effectiveness of the proposed communication-learning principle rather than as a complete validation under realistic aerial dynamics and wireless channels. Future work will incorporate three-dimensional UAV dynamics, obstacles, communication latency, channel interference, quantization, and energy-aware transmission in high-fidelity simulation and real-world robotic platforms.

## Figures and Tables

**Table 1:** Summary of compared methods.

Method	Communication Setting	Message Type	Redundancy Reduction
NoComm-MARL	None	–	–
FullComm-MARL	Global	Dense	No
DistComm-MARL	Local graph	Dense	No
CommNet	Global	Continuous	No
TarMAC	Attention	Targeted	No
SchedNet	Scheduled	Selected	No
GraphComm-MARL	Local graph	Dense	No
KL-IB-MARL	Local graph	Bottleneck	KL
KL-IB-Red	Local graph	Bottleneck	KL
IB-CEMARL	Local graph	Bottleneck	CS

**Table 2:** Main performance comparison under the standard UAV swarm coordination setting. The ↑ and ↓ symbols indicate that higher and lower values are preferred, respectively. The best performance is in bold.

Method	Return ↑	Coverage ↑	Local-Msg. Dep. ↓	Inter-Msg. Dep. ↓
NoComm-MARL	128.4±9.7	62.1±3.8	–	–
FullComm-MARL	171.6±7.4	78.5±2.9	0.72±0.04	0.48±0.03
DistComm-MARL	165.2±8.1	76.9±3.1	0.66±0.05	0.42±0.03
CommNet	158.7±8.9	74.3±3.6	0.69±0.05	0.45±0.04
TarMAC	166.8±7.6	77.4±3.0	0.58±0.04	0.39±0.03
SchedNet	160.5±8.3	75.1±3.4	0.55±0.04	0.37±0.03
GraphComm-MARL	169.1±7.2	78.0±2.8	0.63±0.04	0.40±0.03
KL-IB-MARL	171.9±6.8	79.2±2.5	0.41±0.03	0.31±0.02
KL-IB-Red	177.1±6.1	81.0±2.3	0.31±0.02	0.25±0.02
IB-CEMARL	180.3±5.9	82.6±2.2	0.29±0.02	0.22±0.02

**Table 3:** Ablation study of information-bottleneck components and dependence measures. The ↑ and ↓ symbols indicate that higher and lower values are preferred, respectively. The best performance is in bold.

Variant	Return ↑	Coverage ↑	Local-Msg. Dep. ↓	Inter-Msg. Dep. ↓
w/o compression	170.8±7.0	78.7±2.7	0.67±0.04	0.34±0.03
w/o task relevance	154.2±8.6	72.8±3.5	0.36±0.03	0.24±0.02
w/o redundancy	173.5±6.7	79.5±2.6	0.43±0.03	0.36±0.03
KL-IB-MARL	171.9±6.8	79.2±2.5	0.41±0.03	0.31±0.02
KL-IB-Red	177.1±6.1	81.0±2.3	0.31±0.02	0.25±0.02
IB-CEMARL	180.3±5.9	82.6±2.2	0.29±0.02	0.22±0.02

**Table 4:** Average return under different packet loss rates. The best performance is in bold.

Method	0.0	0.1	0.2	0.3	0.5
DistComm-MARL	165.2	154.7	143.5	131.8	108.6
TarMAC	166.8	157.3	147.1	136.4	113.9
GraphComm-MARL	169.1	160.2	151.6	140.5	119.4
KL-IB-MARL	171.9	164.8	156.9	148.2	128.7
IB-CEMARL	180.3	174.1	166.8	158.9	139.5

**Table 5:** Average return under different communication radii. The best performance is in bold.

Method	$r_{c}=50$	$r_{c}=100$	$r_{c}=150$	$r_{c}=200$
DistComm-MARL	132.6	153.9	165.2	166.1
GraphComm-MARL	137.4	158.5	169.1	169.7
KL-IB-MARL	143.8	163.2	171.9	172.5
IB-CEMARL	151.5	171.6	180.3	181.1

**Table 6:** Generalization to unseen swarm sizes. The best performance is in bold.

Method	N=3	N=5	N=7	N=10
DistComm-MARL	121.4	165.2	176.3	182.7
GraphComm-MARL	125.8	169.1	181.5	188.2
KL-IB-MARL	130.6	171.9	186.4	193.1
IB-CEMARL	137.9	180.3	196.7	205.4

**Table 7:** Computational overhead comparison. The best performance is in bold.

Method	Training Time (h)	GPU Memory (GB)	Inference Time (ms)	Overhead (%)
MAPPO	1.0	3.2	1.8	-
KL-IB-MARL	1.15	3.8	1.9	15.0
IB-CEMARL	**1.35**	**4.1**	**1.9**	35.0

**Table 8:** Performance under different transmitted message dimensions. The best performance is in bold.

Method	$d_{z}=4$	$d_{z}=8$	$d_{z}=16$	$d_{z}=32$
DistComm-MARL	139.8	151.7	165.2	166.5
GraphComm-MARL	145.3	158.9	169.1	170.2
KL-IB-MARL	153.7	164.5	171.9	172.6
IB-CEMARL	166.4	175.2	180.3	181.0

**Table 9:** Message redundancy analysis. The best performance is in bold.

Method	Early Training	Middle Training	Final Policy
DistComm-MARL	0.51	0.47	0.42
GraphComm-MARL	0.49	0.44	0.40
KL-IB-MARL	0.43	0.36	0.31
IB-CEMARL	0.39	0.28	0.22

## Data Availability

The trained models and complete training logs are available from the corresponding author upon reasonable request.

## Appendix A. KDE-Based Estimation of CS Information

Given a mini-batch of paired samples (${\left\{\left(x_{b},z_{b}\right)\right\}}_{b=1}^{B}$), we estimate the Cauchy–Schwarz information as

(A1) $$I_{\mathrm{CS}}\left(X;Z\right)=D_{\mathrm{CS}}\left(p_{XZ},p_{X}p_{Z}\right),$$

using Gaussian kernel density estimation.

Let $K,L\in R^{B\times B}$ denote the kernel matrices:

(A2) $$K_{ij}=\exp\left(-\frac{∥x_{i}-x_{j}{∥}_{2}^{2}}{2\sigma_{x}^{2}}\right),\;L_{ij}=\exp\left(-\frac{∥z_{i}-z_{j}{∥}_{2}^{2}}{2\sigma_{z}^{2}}\right).$$

The KDE-based estimator is given by

(A3) $${\hat{I}}_{\mathrm{CS}}\left(X;Z\right)=-\log\frac{{\hat{V}}_{JM}+\epsilon }{\sqrt{\left({\hat{V}}_{JJ}+\epsilon \right)\left({\hat{V}}_{MM}+\epsilon \right)}},$$

where

(A4) $$\begin{array}{cc} {\hat{V}}_{JJ}\; & =\frac{1}{B^{2}}1^{⊤}\left(K\circ L\right)1, \end{array}$$

(A5) $$\begin{array}{cc} {\hat{V}}_{JM}\; & =\frac{1}{B^{3}}{\left(K1\right)}^{⊤}\left(L1\right), \end{array}$$

(A6) $$\begin{array}{cc} {\hat{V}}_{MM}\; & =\left(\frac{1}{B^{2}}1^{⊤}K1\right)\left(\frac{1}{B^{2}}1^{⊤}L1\right). \end{array}$$

Here, ∘ denotes the Hadamard product, 1 is an all-ones vector, and ϵ is a small constant for numerical stability. The Gaussian kernel bandwidths ($\sigma_{x}$ and $\sigma_{z}$) are selected using the median heuristic. The estimator is differentiable and can therefore be optimized jointly with the MARL objective.
